# Proton Beam Therapy for Sinonasal Neuroendocrine Carcinoma: A Six-Case Series with Dosimetric Comparison and Literature Review

**DOI:** 10.3390/jcm15020477

**Published:** 2026-01-07

**Authors:** Hazuki Nitta, Takashi Saito, Ryota Matsuoka, Shin Matsumoto, Shuho Tanaka, Masahiro Nakayama, Kotaro Osawa, Motohiro Murakami, Keiichiro Baba, Masatoshi Nakamura, Keitaro Fujii, Yoshiko Oshiro, Masashi Mizumoto, Keiji Tabuchi, Daisuke Matsubara, Hideyuki Sakurai

**Affiliations:** 1 Department of Radiation Oncology, University of Tsukuba, Tsukuba 305-8577, Japan; hnitta@pmrc.tsukuba.ac.jp (H.N.); murakami@pmrc.tsukuba.ac.jp (M.M.); baba@pmrc.tsukuba.ac.jp (K.B.); nakamura@pmrc.tsukuba.ac.jp (M.N.); ooyoshiko@hotmail.com (Y.O.); mizumoto@pmrc.tsukuba.ac.jp (M.M.); hsakurai@pmrc.tsukuba.ac.jp (H.S.); 2 Department of Diagnostic Pathology, University of Tsukuba, Tsukuba 305-8577, Japan; rmatsuoka@md.tsukuba.ac.jp (R.M.); matsubarad@md.tsukuba.ac.jp (D.M.); 3 Department of Otolaryngology, Head and Neck Surgery, University of Tsukuba, Tsukuba 305-8577, Japan; smatsumoto-tuk@outlook.jp (S.M.); shuho@md.tsukuba.ac.jp (S.T.); nnmasa@md.tsukuba.ac.jp (M.N.); kotaro.osawa@gmail.com (K.O.); k.fujii@md.tsukuba.ac.jp (K.F.); ktabuchi@md.tsukuba.ac.jp (K.T.)

**Keywords:** sinonasal neuroendocrine carcinoma, sinonasal cancer, head and neck cancer, chemoradiotherapy, proton beam therapy

## Abstract

**Background**: Sinonasal neuroendocrine carcinoma (SNEC) is an extremely rare malignancy, and, to date, no clinical reports have detailed the use of proton beam therapy (PBT) for this disease. The present study describes the clinical courses of patients with SNEC treated with PBT and highlights the advantages of PBT. **Methods**: In this retrospective study, we included patients with pathologically confirmed SNEC without distant metastasis who underwent PBT at our institution between 2006 and 2021. To evaluate the dosimetric advantages of PBT, comparative treatment plans using VMAT were created. **Result**: Six patients with pathologically diagnosed SNEC without distant metastasis were treated with PBT. Multimodal treatment was applied in five patients, including chemotherapy in four cases and surgery in two cases. The median follow-up period was 37.4 months (range: 6.9 to 108.9 months). At the end of the follow-up, three patients were alive without recurrence, while three had died due to the disease. Recurrence occurred in three cases: one local recurrence, one in cervical lymph nodes, and two distant metastases. A late adverse event of Grade 4 vision decrease was observed in one patient on the ipsilateral side. Compared with VMAT, PBT lowered the average brain dose (median 3.3 Gy (RBE) vs. 12.6 Gy), brainstem D2 cc (10.7 Gy (RBE) vs. 34.9 Gy) and contralateral optic nerve D0.1 cc (47.6 Gy (RBE) vs. 63.3 Gy), while doses to the ipsilateral optic pathway were comparable. **Conclusions**: PBT in multimodal treatment achieved feasible local control for SNEC. The dose-sparing effect of PBT was more evident in organs distant from the target, although careful consideration is required for adjacent structures.

## 1. Introduction

Sinonasal neuroendocrine carcinoma (SNEC) is a rare disease that accounts for about 5% of all nasal sinus cancers [1]. Due to its rarity, there is a lack of large-scale prospective data for SNEC and understanding of the disease is based on a limited number of retrospective studies [2,3,4,5,6,7,8,9]. Consequently, the characteristics of SNEC are unclear and a standard treatment strategy has not been established. Therefore, NCCN guidelines suggest that treatment in clinical trials or major medical centers is preferable [10].

Sinonasal cancers, especially advanced cases, are challenging to resect with adequate margins and frequently present as unresectable at the time of diagnosis due to their asymptomatic nature [11]. For these reasons, surgery alone is rarely sufficient for complete treatment, and radiotherapy plays a major role in the treatment strategy [10], including postoperative radiotherapy and definitive radiotherapy for unresectable cases. A meta-analysis has shown that particle therapy is more effective than photon radiotherapy for sinonasal malignancies [12]. In this study, to address the scarcity of reports on proton beam therapy (PBT) for SNEC, we present cases treated with PBT at our center and discuss their clinical courses and treatment strategies, accompanied by dosimetric comparison with photon therapy and a literature review.

## 2. Materials and Methods

### 2.1. Patient Selection and Treatment

Cases of SNEC without distant metastasis that were treated with PBT at our center from 2006 to 2021 were included in the retrospective study. Diagnosis of SNEC was made by pathology and met the following three criteria: (1) positive epithelial markers (cytokeratin AE1/AE3, epithelial membrane antigen (EMA)); (2) positive neuroendocrine markers (synaptophysin, chromogranin A, cluster of differentiation antigen (CD) 56); (3) high malignancy (massive necrosis, numerous cells with mitosis and apoptosis).

**Figure 1:**
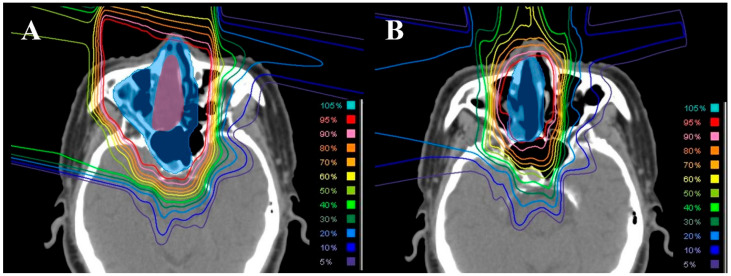
Representative treatment plan for a patient with sinonasal neuroendocrine carcinoma. (**A**) initial plan; (**B**) secondary plan. The red and blue color-filled areas indicate the gross tumor volume and clinical target volume, respectively.

Treatment planning for PBT using the passive scattering method was based on 2.5–5 mm CT scans with the patient immobilized with a thermoplastic mask in the supine position. The VQA 1.7 or 2.0 treatment planning system was used (Hitachi, Tokyo, Japan). The prescribed doses were 2–2.2 Gy (relative biological effectiveness, RBE) per fraction, totaling 60–66 Gy (RBE) for postoperative radiotherapy and 66–70 Gy (RBE) for definitive radiotherapy. The initial clinical target volume (CTV1) encompassed the gross tumor volume (GTV) or tumor bed, including the adjacent nasal cavity and sinuses, and received 40–46 Gy (RBE). Subsequently, the treatment target was shifted to the secondary clinical target volume (CTV2), which was defined as the GTV or tumor bed plus a 5–10 mm margin. An additional 20–24 Gy (RBE) was given to the CTV2. A representative case of this CTV1/CTV2 delineation and the corresponding dose distribution is illustrated in Figure 1. The collimator margin was optimized individually per beam, adding 6–10 mm laterally to the CTV.

The final decision regarding the combination of surgery, chemotherapy and radiotherapy was made at a multidisciplinary team conference, and the treatment plan was determined according to the following principles: Surgery was given the highest priority if the lesion was resectable and the patient was able to tolerate surgery. Induction chemotherapy (IC) was considered for unresectable cases with the aim of improving resectability or reducing the radiation field. Both concurrent and adjuvant chemotherapy were implemented when feasible, including after surgery. The NCCN guidelines were taken into consideration when deciding on a treatment plan [10].

Age, gender, staging based on the 8th edition of the Union for International Cancer Control (UICC) TNM classification, treatment, survival, site of recurrence, salvage treatment, and late adverse events based on Common Terminology Criteria for Adverse Events (CTCAE) ver. 5.0 were retrospectively investigated in each case using the clinical information system of the hospital. In all cases, written consent for use of data for research was obtained prior to treatment.

### 2.2. Dosimetric Comparison with Photon Therapy

To evaluate the dosimetric advantages of PBT, comparative treatment plans using volumetric modulated arc therapy (VMAT) were retrospectively created for patients included in this series. The VMAT plans were designed to cover the same clinical target volumes (CTV1 and CTV2) with the same prescribed total dose as the PBT plans delivered clinically. The VMAT plans were generated on the Raystation treatment planning system (version 14.0; Raysearch Laboratories, Stockholm, Sweden) using a 6-MV photon beam with a two-arc arrangement. A planning target volume (PTV) was created by adding a 5 mm margin to the CTV. The primary planning objective was to ensure that at least 95% of the PTV received 100% of the prescribed dose. For organs at risk (OARs), constraints were set to keep the maximum dose to the brainstem and optic nerves below 50 Gy whenever possible, while the average doses to the brain and eyeballs were kept as low as possible without compromising target coverage. For the dosimetric comparison, we evaluated the following metrics: the average dose to the brain and eyes; the maximum dose to the most-exposed 2 cm^3^ (D2cc) of the brainstem; and the maximum dose to the most-exposed 0.1 cm^3^ of each optic nerve (D0.1cc).

## 3. Results

### 3.1. Clinical Outcomes

The patient characteristics, pathological markers and treatment details of the six cases in the study are shown in Table 1. Four patients were male and two were female. The median age was 72 years (range: 31–84 years). The primary tumor site was the nasal cavity in five cases and the ethmoid sinus in one. Three patients were classified as T4b, involving both the brain and dura mater. Lymph node metastasis was observed in only one case. PBT was administered to two patients as postoperative irradiation and to four as definitive radiotherapy. One case was treated with proton and photon radiotherapy. Surgery was performed in two cases: extensive skull base tumor resection followed by skull base reconstruction in one case, and nasal tumor resection in the other case. Two patients received IC: one with a TPF regimen (docetaxel, cisplatin, and fluorouracil) and the other with cisplatin plus etoposide. Concurrent chemotherapy was administered to three patients: two with cisplatin plus etoposide and one with amrubicin. Subsequently, those who received cisplatin plus etoposide also underwent similar adjuvant chemotherapy.

**Figure 2:**
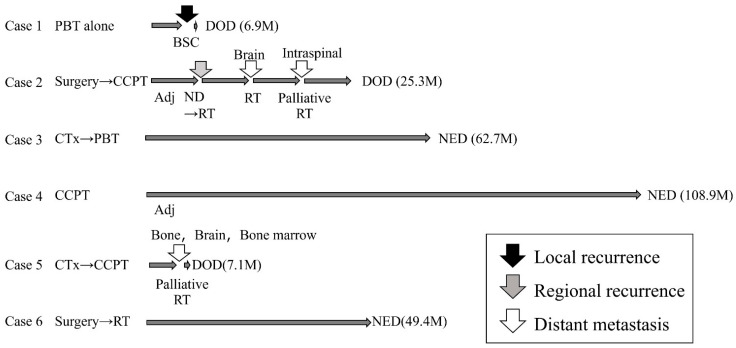
Clinical courses of the 6 cases.

**Figure 3:**
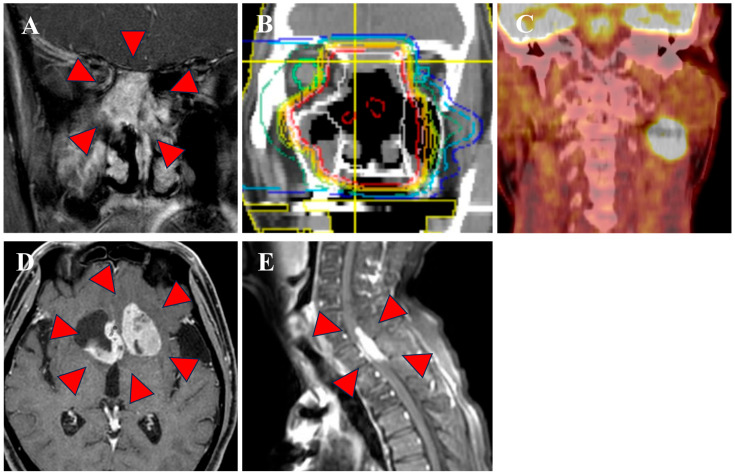
Case of a 72-year old man with SNEC in the right nasal cavity (Case 2). (**A**) Pre-treatment MRI; (**B**) PBT plan; (**C**) Regional recurrence; (**D**) Brain metastasis; (**E**) Intraspinal metastasis; Red triangles indicate primary or metastatic lesions.

The median follow-up period was 37.4 months (range: 6.9 to 108.9 months). At the end of this period, three patients remained alive without recurrence, whereas three patients had died from the disease. Recurrence patterns included one case with local recurrence, one with cervical lymph node recurrence, and two with distant metastases. The clinical course of all 6 cases is shown in Figure 2. The details of three cases with recurrence are as follows. The patient with local recurrence (Case 1), who was deemed inoperable and was of advanced age, underwent definitive radiotherapy with PBT alone. However, six months post-PBT, the patient experienced local re-progression of the disease and ultimately transitioned to best supportive care. Case 2 was a patient with SNEC of the right nasal cavity that was initially treated with surgery and postoperative PBT. Ten months after the initial treatment, right cervical lymph node recurrence occurred. Salvage treatment of neck dissection followed by postoperative photon irradiation at 60 Gy in 30 fractions was performed. However, the patient subsequently developed brain and intraspinal metastases. Despite receiving palliative irradiation for each, the patient died of the disease 25 months after the initial treatment (Figure 3). In contrast, Cases 3 and 4 achieved sustained disease control with combined chemotherapy and PBT, and no recurrence or severe late adverse events have been observed for more than 5 years after the initial treatment. The fifth case (Case 5) underwent definitive PBT with concurrent chemotherapy following IC, but developed distant metastases (bone, brain, bone marrow) 7 months after initial treatment.

Regarding late adverse events, one patient had a vision decrease of Grade 4 on the ipsilateral side (Case 6). This patient received photon radiotherapy to the nasal cavity and left maxillary sinus at a dose of 40 Gy followed by proton boost to the nasal cavity at 20 Gy (RBE). Because the lesion directly abutted the ipsilateral optic nerve, making complete sparing of its function challenging, priority was given to full target coverage; consequently, the entire ipsilateral optic nerve was encompassed by the 60 Gy (RBE) prescription isodose line.

### 3.2. Dosimetric Comparison

**Figure 4:**
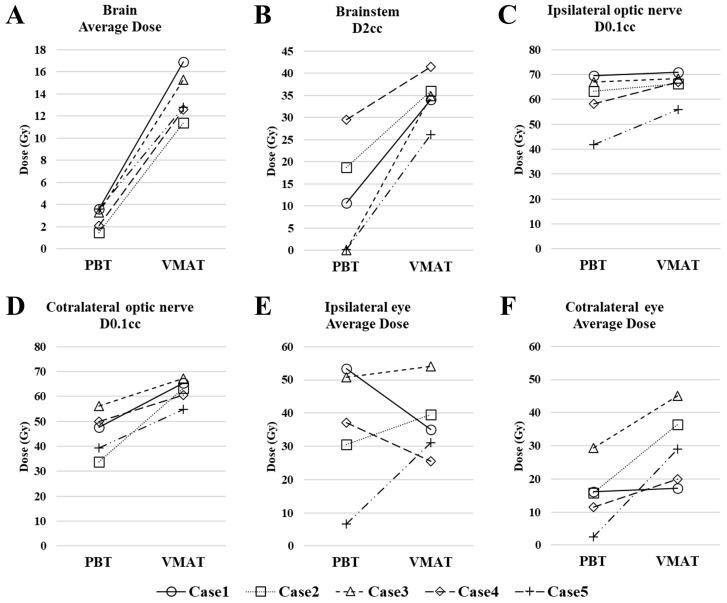
Dosimetric comparison between proton beam therapy (PBT) and volumetric modulated arc therapy (VMAT) plans for organs at risk (OARs). (**A**) brain average dose; (**B**) brainstem D2cc; (**C**) ipsilateral optic nerve D0.1cc; (**D**) contralateral optic nerve D0.1cc; (**E**) ipsilateral eye average dose; (**F**) contralateral eye average dose.

One of the six patients (Case 6) was excluded from this analysis because the patient received combined photon therapy and PBT. For the remaining five cases, the dosimetric comparison between PBT and VMAT plans is presented in Figure 4. The median value of average dose to brain was 3.3 Gy (RBE) (range, 1.5–3.6 Gy (RBE)) with PBT, compared to 12.6 Gy (range, 11.4–16.5 Gy) with VMAT. Similarly, the median brainstem dose (D2cc) was 10.7 Gy (RBE) (range, 0.1–29.5 Gy) with PBT, whereas it was 34.9 Gy (range, 25.5–41.5 Gy) with VMAT. For the contralateral structures, the median dose to the optic nerve (D0.1cc) was 47.6 Gy (RBE) (range, 33.8–56.2 Gy (RBE)) with PBT, compared to 63.3 Gy (range, 54.8–67.2 Gy) with VMAT, and the median average dose to the eye was 15.9 Gy (RBE) (range, 2.5–29.4 Gy (RBE)) with PBT, compared to 29.0 Gy (range, 17.2–45.1 Gy) with VMAT. In contrast, the doses to the ipsilateral optic nerve and eye were comparable between the two modalities.

## 4. Discussion

SNEC is among the rarest cancers of the paranasal sinuses. This rarity has prevented establishment of a treatment strategy for SNEC, and the approach generally follows that for sinonasal SCC [10]. However, distant metastases are more frequent in SNEC than in sinonasal SCC. Pare et al. found distant metastasis in 15.3% of cases of sinonasal SCC [13], whereas the rate in SNEC ranged from 6.7% to 63.6% [2,3,4,5,6,7,8,9]. Therefore, multimodal treatment is often required for SNEC.

Radiotherapy plays an important role in treatment of unresectable sinonasal cancer. Several studies have examined treatment outcomes using radiation therapy for SNEC (Table 2). Most of these patients were treated with multimodal therapy, including radiotherapy, chemotherapy, and surgery. Notably, IC may provide clinically useful information to optimize definitive treatment selection in aggressive sinonasal malignancies. In the largest retrospective series of sinonasal undifferentiated carcinoma treated with a uniform IC-based strategy, response to IC stratified outcomes and informed subsequent local management [14]. Importantly, favorable responders achieved excellent outcomes with definitive chemoradiotherapy, allowing organ-preserving treatment. Because sinonasal tumors can profoundly affect facial function and cosmesis, treatment planning should consider not only functional preservation but also maintenance of appearance whenever feasible. Conversely, poor responders to IC may derive greater benefit from a surgery-based approach when feasible, whereas outcomes with definitive chemoradiotherapy appear unfavorable. Therefore, prioritizing disease control and survival over organ preservation may be warranted in this setting [14].

Moreover, the optimal radiation dose for SNEC remains an important issue. Generally, a total dose of >65 Gy is considered to be preferable for sinonasal SCC; however, toxicities to adjacent critical structures make dose escalation difficult with photon radiotherapy [15]. We have used PBT for head and neck cancer for more than 20 years. A proton beam has an energy peak (the Bragg peak) that permits delivery of a higher dose while avoiding adjacent structures. We previously reported the results of PBT for locally advanced unresectable sinonasal SCC, for which all patients received ≥70 Gy (RBE) for definitive treatment [16]. Given the dismal prognosis of unresectable sinonasal SCC, dose escalation beyond conventional levels may be considered when OAR constraints can be respected to maximize local control. In contrast, due to the extreme rarity of SNEC, an optimal radiation dose has not been established. Therefore, in the present cohort, we treated patients using a conventional dose–fractionation range commonly employed for head and neck malignancies. All patients received a delivered dose ≥60 Gy (RBE), with 60–66 Gy (RBE) postoperatively and 66–70 Gy (RBE) for definitive treatment (Table 1). In our cohort, except for one patient treated with PBT alone, local control was achieved in five patients who received multimodality therapy with surgery and/or chemotherapy. Thus, within a multimodality strategy, the dose–fractionation range used in this study appears feasible in terms of balancing tumor control and toxicity. However, given the limited sample size, further studies are required to define the optimal dose for SNEC.

In our study, a comparison of treatment plans revealed that PBT reduced the dose to surrounding normal organs compared to VMAT. This is consistent with previous research which has shown that PBT can lower the dose compared to VMAT for OARs distant from the target, such as the brain, brainstem, and contralateral optic nerve [17]. On the other hand, the same study reported that the superiority of PBT was limited for organs close to the target, such as the ipsilateral optic nerve and eye [17]. This limitation was also evident in our analysis, as we observed no substantial dosimetric advantage for the ipsilateral optic nerve and eye. These findings underscore the significant challenge of sparing OARs immediately adjacent to the target, even with PBT. Accordingly, in our cohort, in one case in which the lesion directly abutted the ipsilateral optic nerve, the risk of visual impairment was discussed upfront and target coverage was prioritized over optic nerve sparing. The occurrence of a severe late event (ipsilateral blindness) in this case underscores the importance of thorough informed consent and shared decision-making when determining the treatment strategy for lesions adjacent to the optic nerve or eye.

The limited number of cases and retrospective design prevent definitive conclusions in this study. The heterogeneity of the treatment protocols, including the presence or absence of surgery and variations in chemotherapy regimens among cases, is a notable limitation. Therefore, the potential synergistic effects of multimodal treatment cannot be excluded, and the observed clinical outcomes may not be solely attributable to PBT. In addition, our PBT–photon dosimetric comparison was based on treatment planning only and was not linked to clinical outcomes. Thus, the impact of dose reduction on OAR cannot be determined from this study. SNEC is a rare disease that was first described only 40 years ago [18] and the classification of SNEC has been refined to distinguish it from other similar carcinomas [2]. Therefore, a treatment strategy for SNEC may be best established through accumulation of findings from a series of small studies.

## 5. Conclusions

PBT in multimodal treatment achieved feasible local control for SNEC at doses over 60 Gy (RBE) while reducing the dose to surrounding OARs. This advantage was more pronounced for organs distant from the target; however, protecting structures immediately adjacent to the target remains challenging.

## Figures and Tables

**Table 1:** Characteristics, pathological markers, treatments, and outcomes of patients with SNEC.

Case	Age	Sex	Stage		Reg Ion	Size	Pathological Markers									Treatment	Dose	Recurrence	Out Come	Adverse Event
			T	N		(cm)	Mitosis (/10 HPF)	Apoptosis (/10 HPF)	AE1/AE3	EMA	S-100	SYP	CGA	CD56	Ki67 (%)	(Regimen)	(Gy (RBE))			
1	84	M	4b	0	Nasal cavity	6.7	25	≥50	1	NA	NA	1	NA	1	90	PBT alone	70	Local	DOD	-
2	72	M	2	0	Nasal cavity	2.5	40	≥50	2	2	0	3	0	3	NA	Surgery +CCPT (EP) +Adj (EP)	66	Cervical LN Distant	DOD	-
3	62	F	4b	0	Ethmoid sinus	4.5	20	≥50	0	2	0	2	0	2	70	Induction (TPF) + PBT	66	-	NED	-
4	76	M	1	0	Nasal cavity	4.1	25	≥50	NA	3	0	2	1	3	100	CCPT (EP) +Adj (EP)	66	-	NED	-
5	31	F	4b	2b	Nasal cavity	5.6	20	≥50	1	NA	NA	3	2	3	NA	Induction (EP) + CCPT (AMR)	60	Distant	DOD	-
6	81	M	3	0	Nasal cavity	7.2	8	≥50	1	0	0	1	2	3	NA	Surgery + RT (Photon + Proton)	Photon: 40 PBT: 20	-	NED	Vision decreased Grade 4

HPF: high power field, AE1/AE3: cytokeratin AE1/AE3, EMA: epithelial membrane antigen, S-100: S-100 protein, SYP: synaptophysin, CGA: chromogranin A, CD: cluster of differentiation antigen, RBE: relative biological effectiveness, NA: not available, 0: negative, 1: weak positive, 2: moderate positive, 3: strong positive, PBT: proton beam therapy, RT: radiotherapy, Photon: photon radiotherapy, CCPT: concurrent chemo-proton therapy, Adj: adjuvant chemotherapy, Induction: induction chemotherapy, EP: cisplatin plus etoposide, TPF: TPF regimen (docetaxel, cisplatin, and fluorouracil), AMR: amrubicin, LN: lymph node, DOD: died of disease, NED: no evidence of disease.

**Table 2:** Previous reports of sinonasal neuroendocrine carcinoma.

Author	Year	Number of Patients	Radiotherapy Modality	Median Observation Period (Months)	Treatment *				Overall Survival	Local Recurrence	Neck Recurrence	Distant Metastasis	Metastasis Region
					Surgery	Chemotherapy	Radiotherapy	Radiation Dose (Gy)					
Keilin et al. [2]	2022	13	Photon	-	5	8	11	43.5–70	74.6% (5y)	7.7%	7.7%	7.7%	Lung
				6.3–138.2									
Sekine et al. [3]	2021	7	Photon	-	2	7	6	40–60	28.6% (5y)	14.3%	14.3%	42.9%	Brain, Liver, Bone
				2–192									
Turri-Zanoni et al. [4]	2017	22	Photon	22	22	>10	20	50–66	42.6% (5y)	13.6%	18.2%	63.6%	Brain, Liver, Bone, Lung, Kidney, Pancreas
van der Laan et al. [5]	2013	12	Photon	30.5	8	0	9	30–70.2	41.7%	16.7%	16.7%	8.3%	-
Mitchell et al. [6]	2012	28	Photon	48.6	13	16	16	-	66.9% (5y)	21.4%	25%	14.3%	-
Likhacheva et al. [7]	2011	20	Photon	60	15	13	17	>60	-	10%	15%	10%	Dura mater, Pia mater
Wang et al. [8]	2008	10	Photon	74.5	10	4	5	-	70% (5y)	20% (5y)	0%	40% (5y)	Brain, Liver, Bone, Lung
Babin et al. [9]	2006	21	Photon	12	11	12	13	30–78	-	19%	14.3%	19%	Brain, Liver, Bone
Current study	2025	6	Proton	37.4	2	4	6	60–70 (RBE)	50% (4y)	17%	17%	33%	Brain, Bone, Intraspinal, Bone marrow

* Total number of patients, RBE: relative biological effectiveness.

## Data Availability

The datasets used and/or analyzed during the current study are available from the corresponding author on reasonable request.

## Abbreviations

The following abbreviations are used in this manuscript:

SNEC	sinonasal neuroendocrine carcinoma
PBT	proton beam therapy
RBE	relative biological effectiveness
IC	induction chemotherapy
EMA	epithelial membrane antigen
CD	cluster of differentiation antigen
GTV	gross tumor volume
CTV	clinical target volume
UICC	Union for International Cancer Control
CTCAE	Common Terminology Criteria for Adverse Events
VMAT	volumetric-modulated arc therapy
PTV	planning target volume
OAR	organs at risk
D2cc	most-exposed 2 cm^3^
D0.1cc	most-exposed 0.1 cm^3^
